# Herbal formulations, Product Nkabinde and *Gnidia sericocephala*, exhibit potent *in vitro* activity against HIV-1 infection

**DOI:** 10.3389/fphar.2025.1618187

**Published:** 2025-07-02

**Authors:** Khanyisile Mngomezulu, Paradise Madlala, Siphathimandla Authority Nkabinde, Magugu Nkabinde, Mlungisi Ngcobo, Nceba Gqaleni

**Affiliations:** ^1^ Traditional Medicine Laboratory, School of Nursing and Public Health, Howard College, University of KwaZulu-Natal, Durban, South Africa; ^2^ Africa Health Research Institute (AHRI), Durban, South Africa; ^3^ HIV Pathogenesis Programme, University of KwaZulu-Natal, Durban, South Africa; ^4^ Ungangezulu, Dundee, South Africa; ^5^ Faculty of Health Sciences, Durban University of Technology, Durban, South Africa

**Keywords:** HIV-1, African traditional medicine, anti-HIV-1 activity, TZM-bl cell line, PBMCs

## Abstract

**Background:**

While antiretroviral therapy (ART) has transformed HIV-1 into a manageable chronic illness, its long-term affordability and accessibility remain major challenges in resource-limited settings. Additionally, adverse side effects can compromise treatment adherence and effectiveness. These limitations highlight the need for novel, affordable therapeutic alternatives. In this study, we evaluated the anti-HIV-1 activity of Product Nkabinde (PN), a traditional herbal formulation comprising four plant extracts, and *Gnidia sericocephala* (*G. sericocephala*), to assess their potential as alternative or complementary therapies.

**Methods:**

HIV-1 subtype B and subtype C viral stocks were produced by transfecting HEK293T cells with envelope plasmids and an *env*-deficient HIV-1 backbone vector using polyethylenimine. TZM-bl cells were treated with PN and *G. sericocephala* extracts, alone or combined with antiretrovirals (AZT, raltegravir, maraviroc, amprenavir), then infected with the viruses. Viral infectivity was measured using the luciferase assay, and results were validated in peripheral blood mononuclear cells (PBMCs) using HIV-1 p24 ELISA.

**Results:**

The PN extract exhibited a dose-dependent antiviral effect, with the optimal concentration achieving 93% and 96% inhibition of HIV-1 subtype B and C, respectively, in TZM-bl cells, comparable to AZT. In HIV-1 infected PBMCs, treatment with AZT, PN, or *G. sericocephala* resulted in a sustained reduction of p24 antigen levels over 11 days compared to untreated controls. While NL4.3 showed partial inhibition (p24 levels >20,000 pg/mL), strains CM070P.1, YU2, and CM019P.1.2 exhibited consistently low p24 production levels (<20,000 pg/mL), indicating strain-dependent antiviral activity. PN, combined with maraviroc inhibited YU2 replication by 81.3% (p = 0.0361), while combinations with raltegravir and AZT suppressed subtype C strains CM070P.1 and CM019P.1.2 by 98.7% (p = 0.0083) and 99% (p = 0.0428), respectively, compared to either PN or the antiretroviral alone. *Gnidia sericocephala* combined with AZT inhibited NL4.3 by 80.3% (p = 0.0105), and its combinations with maraviroc, raltegravir, and amprenavir suppressed CM070P.1 replication by 87% (p = 0.0093), 86% (p = 0.0168), and 90% (p = 0.0006), respectively, relative to either test agent alone. Fractional inhibitory concentration index (FICI) analysis indicated no synergistic or antagonistic interactions.

**Conclusion:**

Thus, this current data suggests that PN and *G. sericocephala* possess anti-HIV-1 activity.

## 1 Introduction

Human immunodeficiency virus type 1 (HIV-1) remains a major global health burden, with ongoing challenges in prevention, treatment accessibility, drug resistance, and the absence of an effective vaccine. By the end of 2023, approximately 39.9 million people were living with HIV-1 (PLWH) globally, with 1.3 million new infections and 630,000 AIDS-related deaths reported. Sub-Saharan Africa bears the highest burden, accounting for approximately 25.6 million PLWH, including 7.8 million in South Africa alone (UNAIDS, 2024). The introduction of antiretroviral therapy (ART) has dramatically improved the prognosis of PLWH, transforming HIV-1 into a manageable chronic condition and significantly reducing mortality (Matsuda and Maeda, 2024; Palshetkar et al., 2020; Peltzer et al., 2011).

Although ART effectively suppresses viral replication and reduces plasma viral load to undetectable levels, it cannot eliminate the virus due to the persistence of latent, replication-competent reservoirs established early during acute infection (Matsuda and Maeda, 2024; Kent et al., 2013; Geeraert et al., 2008; Finzi et al., 1997). As a result, lifelong ART is required, which poses challenges including drug-induced toxicity, chronic immune activation, neurocognitive impairment, and the emergence of drug-resistant strains (Kankara et al., 2022; Ta et al., 2022; Deeks et al., 2013; Peterson et al., 2011; Montessori et al., 2004). In resource-limited settings, barriers to ART further complicate long-term management (Sagaon-Teyssier et al., 2016). These limitations necessitate the development of novel therapeutic strategies, particularly plant-derived compounds with improved efficacy, novel mechanisms of action, favorable pharmacokinetics, and reduced toxicity (Palshetkar et al., 2020; Kurapati et al., 2016; Mandal et al., 2020).

Natural products, especially those derived from plants, have played a central role in drug discovery and remain a rich source of structurally diverse and biologically active compounds (Mandal et al., 2020; Palshetkar et al., 2020; Kurapati et al., 2016). Recognizing the historical importance of natural products in drug discovery, the World Health Organization (WHO) emphasized the evaluation of medicinal plants for HIV/AIDS management as early as 1989 (Kurapati et al., 2016; WHO, 1989). South Africa is particularly rich in plant biodiversity, with over 3,000 species used in traditional medicine (Van Wyk and Gericke, 2000; Scott et al., 2004), many of which have demonstrated anti-HIV-1 activity (Terefe et al., 2022). Plant-derived compounds, including alkaloids, coumarins, carbohydrates, flavonoids, lignans, phenolics, quinines, phospholipids, terpene, and tannins, have shown inhibitory HIV-1 activity at almost every step of the viral life cycle (Terefe et al., 2022; Jiang et al., 2010).

Traditional, complementary, and alternative medicine (TCAM) is widely used by PLWH, especially in rural South Africa, where more than 80% of the population relies on traditional healers for managing infectious diseases (Audet et al., 2021; Abdullahi, 2011; Malangu, 2007; King, 2000). Studies have shown that up to 75% of PLWH in South Africa incorporate traditional or complementary remedies alongside ART to manage HIV/AIDS symptoms such as fatigue, nausea, depression, insomnia, and dermatological conditions (Peltzer et al., 2011; Malangu, 2007; Duggan et al., 2001; Standish et al., 2001). A clinical study by Tshibangu et al. (2004) found that an unidentified South African traditional medicine significantly reduced plasma viral load and increased CD4^+^ T cell counts in 33 PLWH compared to standard care alone without the herbal supplement.

Numerous medicinal plants used by traditional health practitioners (THPs) for the treatment of HIV/AIDS and related conditions have been identified and evaluated, including *Hypoxis hemerocallidea* (African potato) and *Sutherlandia frutescens* (Klos et al., 2009; Mills et al., 2005). A study screened five aqueous and ethanolic South African medicinal plant extracts for their anti-HIV-1 activity in HIV-1 infected CEM.NK®-CCR5 cells and ethanolic extracts of *Leonotis leonurus* and *Bulbine alooides* showed the greatest anti-HIV-1 activity (Klos et al., 2009). Similarly, a study by Terefe et al. (2022) evaluated the cytotoxicity and anti-HIV activity of crude extracts from three plants, namely *Croton macrostachyus, Croton megalocarpus,* and *Croton dichogamus* against the HIV-1_IIIB_ strain in MT-4 cell line. The leaf extract of *Croton megalocarpus* and aerial extract of *Croton dichogamus* exhibited potent anti-HIV-1 activities with reduced toxicity and high selectivity index values. In addition, another study demonstrated that two phorbol ester derivatives, NPB-11 (12-O-methoxymethylphorbol-13-decanoate) and NPB-15 (12-O-benzyloxymethylphorbol-13-decanoate) suppressed HIV-1_IIIB_ replication in MT-4 cell line and blocked CXCR4 and CCR5 co-receptor in PBMC cells measured by p24 antigen ELISA assay (Zhong et al., 2005). Furthermore, an *in vitro* study assessed the anti-HIV-1 activity of traditional medicines obtained from local THPs using MT-4 cells. Three of the five tested medicines (*Ugambu, Ihashi, and Product Nene*) demonstrated promising anti-HIV activity compared to AZT antiretroviral drugs (Gqaleni et al., 2012).

Given this context, we examined the anti-HIV-1 properties of African traditional medicine, referred to as Product Nkabinde (PN) in this manuscription. This traditional medicine is a polyherbal formulation from four known medicinal plants, namely *Sclerocarya birrea subsp. afra (Anacardiaceae), Gnidia sericocephala (Meisn.) gilg ex Engl. (Thymelaeaceae), Senna italica subsp. italica (Fabaceae), and Pentanisia prunelloides (Klotzsch ex Eckl. & Zeyh.) Walp. subsp. prunelloides (Rubiaceae)* obtained from THPs (Mr. S and M Nkabinde) who reside in the rural parts of KwaZulu-Natal. A previous study demonstrated PN’s *in vitro* immunomodulatory effects on healthy PBMCs, suggesting potential anti-inflammatory and immune enhancing activity (Setlhare et al., 2024). Therefore, in this study, we sought to assess the *in vitro* and *ex vivo* anti-HIV-1 activity of the aqueous crude extracts of PN and the single-plant ethanolic extract of *G*. *sericocephala*.

## 2 Methods

### 2.1 Ethics statement

The University of KwaZulu-Natal Ethics Committee provided ethical approval for this study and consent processes (BREC approval number: BREC/00001973/2020).

### 2.2 Plant material and preparation of plant extract

This is a Type C extract consisting of botanical drugs and their extracts derived from lesser-studied species and the drugs derived from them, which are not included in a national or regional pharmacopeia and are not used commercially at an international level. The traditional medicine was obtained from THPs, Mr S Nkabinde and Mr M Nkabinde. The medicinal plant parts, namely, *Sclerocarya birrea* stem/leaf, *Gnidia sericocephala* roots, *Senna italica* roots, and *Pentanisia prunelloides* roots were collected in Tugela Ferry in Msinga, KwaZulu-Natal by the healers. The plants were verified and classified by Ms Magda Nel, an ethnobotanist at the H.G.W.J Schweickerdt Plant Herbarium at the University of Pretoria where the voucher specimens were deposited as shown in Table 1 (Setlhare et al., 2024). In the laboratory, PN was filter-sterilized and freeze-dried using a Benchtop Freeze Dryer (VirTis, Sp Scientific, Warminster, PA, United States) to form a powder and stored at −20°C until needed for other experiments. A water: ethanol mixture extract of *G. sericocephala* was manufactured by Afriplex Pharmaceuticals (https://afriplex.co.za) in Paarl, South Africa, through spray-drying technology. For extract preparation, PN (10 mg/mL) was weighed and dissolved in phosphate-buffered saline (PBS) (Thermo Fisher Scientific, Carlsbad, CA, United States) and filter sterilized. *Gnidia sericocephala* was weighed (10 mg) and first dissolved in 2% dimethylsulfoxide (DMSO) (Sigma-Aldrich, St Louis, MO, Untied States) then 10 mL of PBS to obtain a final concentration of 10 mg/mL.

**TABLE 1:** Plant extract information and their voucher specimen number (Setlhare et al., 2024).

Plant name	Plant part used	Voucher specimen number
Sclerocarya birrea	Stem and leaf	126589
Gnidia sericocephala	Roots	126590
Senna italica	Roots	126591
Pentanisia prunelloides	Roots	126592

A patent for PN has been filed in South Africa with reference number 2023/03587 and a global patent with reference number PCT/IB 2024/052,529 has also been filed for PN. Setlhare et al. (2024) and Tembeni et al. (2022) have described the UPLC-HRMS and HPLC analysis of PN and *G. sericocephala* phytochemicals, respectively.

### 2.3 Compounds

Zidovudine (AZT), efavirenz, raltegravir, maraviroc and amprenavir were obtained from the National Institute of Health (NIH). These compounds were dissolved in DMSO (not more than 1%) and diluted in PBS. Each drug was reconstituted to prepare a stock of 20 mg/mL Phytohemagglutinin (PHA) purchased from Sigma-Aldrich (St Louis, MO, United States).

### 2.4 Cell culture and reagents

The HEK293 cells are a derivative of the human embryonic kidney cells (HEK293T), established by the expression of a temperature-sensitive SV40 T-antigen mutant (Bae et al., 2020; DuBridge et al., 1987). The expression of the T-antigen permits plasmids that carry the SV40 origin of replication to replicate when transfected into the cell (DuBridge et al., 1987). TZM-bl cell line, also known as the JC.53bl-13 cell line, is a genetically modified HeLa cervical cancer cell line engineered to express CD4, CCR5, and CXCR4 (Platt et al., 1998) to allow for HIV-1 entry. The cells harbor integrated reporter genes for firefly luciferase and β-galactosidase under the control of the HIV-1 long terminal repeat (LTR), allowing quantification of infection through luminescence or colorimetric assays (Xing et al., 2016; Wei et al., 2002). Both cell lines were obtained from the National Institute of Health (NIH AIDS Research and Reference Reagent Programme) and maintained in Dulbecco’s Modified Eagle Medium (DMEM) (Gibco, Grand Island, NY, United States) supplemented with 10% heat-inactivated fetal bovine serum (FBS) (Gibco, Grand Island, NY, United States), 100 mL penicillin-streptomycin (Thermo Fisher Scientific, Carlsbad, CA, United States), and 25 mM Hepes buffer (Thermo Fisher Scientific, Carlsbad, CA, United States) at 37°C in a 5% carbon dioxide (CO_2_) atmosphere as previously described (Jiang et al., 2010).

Peripheral blood mononuclear cells were obtained from participants enrolled in the Females Rising through Education, Support, and Health (FRESH) cohort, established in 2012 in Durban, KwaZulu-Natal, South Africa. The FRESH study is an ongoing prospective cohort that recruits HIV-1 negative adolescent females aged 18–23 years who are at high risk of HIV-1 acquisition. In addition to biomedical monitoring, the study integrates socioeconomic empowerment interventions aimed at addressing structural factors contributing to HIV-1 vulnerability. Longitudinal blood and mucosal samples are collected pre- and post-enrolment to facilitate the detection and characterization of acute HIV-1 infection (Dong et al., 2018). To date, over 2,500 participants have been enrolled, with 95 cases of acute HIV-1 infection identified. Cryopreserved PBMCs obtained at baseline before HIV-1 acquisition, were made available from 6 study participants. Frozen PMBCs were thawed and cultured in complete growth medium composed of RPMI 1640 containing 10% FBS, 100 μg/mL penicillin-streptomycin, 1% L-glutamine, 1% Hepes, and 10 µg (10 μg/mL) recombinant human IL-2, CF (Roche, Germany) and activated using 5 μg/mL phytohemagglutinin (PHA).

### 2.5 Cytotoxicity assay

To determine the safety profile of the traditional medicines (PN and *G. sericocephala*) on PBMCs and TZM-bl cell line, a cell viability assay was performed using the CellTiter-Glo^®^ Luminescent Cell Viability Assay according to the manufacturer’s protocol (Promega, Madison, United States). Briefly, PBMCs were seeded at 1 × 10^6^ cells/mL in 24-well microtiter plates in R10 medium and treated with extracts at defined concentrations of PN (10, 50, 100, 200, 300, and 325 μg/mL), and *G. sericocephala* (10, 50, 100, and 106 μg/mL) in triplicates and incubated for 24 h at 37^0^C at 5% CO_2_. Untreated cells (cells in cell culture medium) served as the negative controls.

Following incubation, 100 µL of treated/control PBMCs were transferred into a white 96-well opaque plate, and 100 µL of CellTiter-Glo^®^ reagent was added to each well and the plate was placed in the orbital shaker for 2 min to induce cell lysis. The luminescence was then measured using the Victor Nivo Multimode plate reader (PerkinElmer, Massachusetts, United States). Assays were performed in triplicate from three independent experiments and the 50% cytotoxicity concentrations (CC50) values were generated using GraphPad Prism software (Version 10, GraphPad, La Jolla, CA).

TZM-bl cells were seeded overnight (16–18 h) into a 96-well, flat bottom tissue culture plate at a density of (1 × 10^4^) cells per well in 100 μL of supplemented DMEM growth media. The following day, the cell media was removed and replaced with 200 μL of fresh complete DMEM growth media and treated with varying concentrations of PN and *G. sericocephala* as per the PBMCs above. The treated cells were incubated at 37°C in a 5% CO_2_ incubator for 24 h. Following 24-h incubation, the cells were trypsinized and 100 µL was transferred into the white 96-well opaque plate. The number of viable TZM-bl cells was determined using the CellTitre-Glo reagent procedure as mentioned above for PBMCs, and the CC50 concentration was calculated.

### 2.6 Transformation of plasmids into JM109 *Escherichia coli* competent cells and proviral plasmid construction

HIV-1 env expression plasmids (pSVIII-NL4.3, pSVIII-CM070P.1, pSVIII-YU2, and pSVIII-CM019P.1.2) and the pBR43-ΔEnv-NefSF2GFP backbone were provided by Dr. Katlego Sojane (HIV Pathogenesis Programme, University of KwaZulu-Natal, Durban, South Africa). Each of the five plasmids (pSIII-YU2, pSVIII-NL4.3, pSVIII-CM070P.1, pSVIII-CM019P.1.2, and pBR43-ΔEnv-NefSF2GFP) was propagated by transforming it into the bacterial strain JM109 *Escherichia coli* (*E. coli*) competent cells (Promega, United States, Madison) individually, as per the manufacturer’s instructions. Briefly, 2 µL of each of the plasmid DNA was independently added to its respective 50 µL of JM109 *E. coli* competent cells and immediately placed on ice for 30 min and heat shock the cells at 42°C. Thereafter, 250 µL of super optimal broth with catabolic repression (SOC) media was added into the transformation mixture and placed in the shaking incubator for 60 min at 37°C at a speed of 230 rpm. In an agar plate containing ampicillin, 100 µL of the transformation mixture was spread on the plate, left to dry, and then the plates were placed in the 37°C incubator for 16 h. The following day, colonies were cultured in lysogeny broth (LB) containing Ampicillin antibiotic and incubated for 16 h at 37°C while shaking at 230 rpm.

### 2.7 Plasmid DNA purification

The plasmid DNA was purified using the GeneJET plasmid miniprep kit (Thermo Fisher Scientific, Carlsbad, CA, United States) as per the manufacturer’s instructions. After purification, the plasmid DNA quality was assessed by using the NanoDrop™ 2000 software version 1.6.198 (Thermo Fisher Scientific, Carlsbad, CA, United States). The purified PCR product was restricted using BamHI-HF and Kpn1 to confirm the inserts (Thermo Fisher Scientific, Carlsbad, CA, United State). The positive colonies were confirmed by using 1% agarose gel electrophoresis.

### 2.8 Pseudovirus production in HEK293T cells and titration

The HEK293T cell line was used for pseudovirus production as previously described (Ozaki et al., 2012; Li et al., 2005). Briefly, viral stocks for HIV-1 strains NL4.3 (subtype B, using the CXCR4 coreceptor), CM070P.1 (subtype C, using the CXCR4 coreceptor), YU2 (subtype B, using the CCR5 coreceptor) and CM0190P.1.2 (subtype C, using the CCR5 coreceptor) were produced by co-transfecting exponentially dividing 293T cells (0.3 × 10^6^ cells/mL growth medium in a T-75 culture flask) with 0.8 µg envelope-expressing plasmids (pNL4.3 or, pCM070P.1 or, pYU2 or, and pCM0190P.1.2) and 2.4 µg of an *env*-deficient HIV-1 backbone vector (pBR43-ΔEnv-NefSF2GFP) using polyethyleneimine (PEI) transfection reagent (Sigma-Aldrich, St Louis, MO, United States). The cell culture supernatants containing pseudotype viruses were harvested 48 h post transfection by filtration (0.45 µm) and then stored in 1 mL aliquots at −80°C until further use. The 50% tissue culture infective doses (TCID50) titer of each pseudovirus aliquot was determined using a TZM-bl luciferase-based assay. For TCID50 measurement, fivefold serial dilutions of pseudovirus were made in septuplicate wells in 96-well culture plates in a total volume of 100 µL growth medium for a total of six dilution steps. Trypsinized TZM-bl cells (10,000 cells in 100 µL of growth medium containing 25 μg/mL DEAE-dextran) were added to each well, and the plates were incubated at 37°C in a humidified 5% CO2 for 48 h. The viral titers were determined by measuring the luciferase activity using the Bright-Glo luciferase reagent, according to the manufacturers’ instructions (Promega, Madison, WI, United States). The plate was read with the Victor Nivo Multimode plate reader (PerkinElmer, Massachusetts, United States). The wells that produced relative luminescence units (RLU) > 3X background were scored as positive. Results were analyzed using GraphPad Prism10. The TCID50 was determined by the virus dilution that was able to produce 20,000–50,000 RLUs to ensure a standardized virus dose in the assay (Li et al., 2005).

### 2.9 Luciferase-based virus inhibition assays in TZM-bl cell line

The anti-HIV activity of PN and *G. sericocephala* extracts was assessed using the TZM-bl luciferase-based assay as previously described (Li et al., 2005). Briefly, 50 µL TCID50 of each virus was incubated with serially diluted (eight dilutions, threefold stepwise) concentrations of PN (starting from 32.5 μg/mL), *G. sericocephala* (106.4 μg/mL) or zidovudine (AZT, 262.5 μg/mL) in triplicate for 1 h at 37°C, humidified 5% CO_2_ atmosphere, in a total volume of 150 µL DMEM in 96-well plates. Following incubation, freshly trypsinized cells were added per well and incubated for 48 h under the same conditions. Each experiment included a positive control consisting of untreated TZM-bl cells infected a corresponding virus, referred to as virus-only control (cells + virus) while the negative control was untreated and uninfected TZM-bl cells, referred to as cell-only control (uninfected). Infectivity of the virus was quantified using Bright-Glo™ luciferase assay (Promega, Madison, WI, United States) according to the manufacturer’s protocol. Luminescence was measured with a Victor Nivo Multimode Plate Reader (PerkinElmer, Waltham, MA, United States). The half-maximal inhibitory concentration (IC_50_) was defined as the concentration of compound (PN, *G. sericocephala*, or AZT) required to reduce chemiluminescence units by 50% compared to virus control wells. The HIV-1 inhibition percentage was expressed using this calculation:

$$\%\text{ inhibition of HIV}‐1=\frac{\left.\left(1-(\text{Average}\;\;\;\;\;\text{treatment}-\text{Average}\;\;\;\;\;\text{cell control}\right)\right)}{\left.\left(\text{Average}\;\;\;\;\;\;\text{virus control}\;\;\;\;\;\;\text{control}-\text{Average}\;\;\;\;\;\text{cell control}\right)\right.}\times 100$$

The CC50 values represent the concentration of the TM extract or drug that causes 50% toxicity on each cell line. The IC_50_ values represent the concentration of each treatment that decreases the viral titer by 50%. These values were used to calculate the selectivity index (SI) of each treatment using the following formula:

$$\text{Selectivity index}\left(\text{SI}\right)=\frac{{CC}_{50}}{{IC}_{50}}$$

### 2.10 HIV-1 inhibition in PBMCs using p24 ELISA assay

For confirmation of the anti-HIV-1 activity of crude extracts, the inhibitory effect of PN and *G. sericocephala* extracts on HIV-1 subtype B and C replication in primary cells was assessed by quantifying p24 antigen levels. Briefly, cryopreserved peripheral blood mononuclear cells (PBMCs) were thawed and rested for 2 h at 37°C in a humidified 5% CO_2_ atmosphere in complete growth medium. Rested cells were adjusted to 2 × 10^6^ cells/mL and stimulated with 5 μg/mL phytohemagglutinin (PHA) for 48 h. Activated PBMCs (1 × 10^6^ cells in 100 µL R10) were then infected with HIV-1 subtype B (NL4.3 and YU2) or subtype C (CM070P.1 and CM019P.1.2) and treated with PN, *G. sericocephala*, or AZT at their CC10 concentrations (corresponding to 90% cell viability), in a final volume of 500 µL. Viral inoculation was performed by spinoculation at 12,000 × g for 30 min, followed by incubation at 37°C for 72 h. Every 3 days, 200 µL of culture supernatant was harvested by centrifugation (1800 rpm, 5 min), replaced with fresh pre-warmed R10 medium containing 10 μg/mL IL-2, and cultures were maintained at 37°C. Collected supernatants were aliquoted and stored at −80°C for quantification of HIV-1 p24 levels using the QuickTiter™ Lentiviral Quantification Kit (Cell Biolabs Inc., San Diego, CA, United States), according to the manufacturer’s instructions.

### 2.11 Combination of TM and antiretroviral drugs assay

The cytotoxicity of individual drugs (AZT, raltegravir, maraviroc, and amprenavir) was evaluated using the CellTiter-Glo^®^ Luminescent Cell Viability Assay. Furthermore, antiretroviral drugs and traditional medicine extracts were combined at a 1:1 ratio to examine potential drug interactions. The IC_50_ concentrations of each drug and traditional medicine were used individually or in combination to treat TZM-bl cells infected with HIV-1 strains (NL4.3, CM070P.1, YU2, and CM019P.1.2) (Table 2). TZM-bl cells (1 × 10^5^ cells/well) containing 25 μg/mL DEAE-dextran were seeded in 96-well plates and incubated at 37°C with 5% CO_2_ for 48 h. Following incubation, the culture medium was replaced with Bright-Glo™ luciferase assay reagent (Promega, Madison, WI, United States), and the plates were incubated for 2 min. Cell lysates were transferred to black 96-well plates and the luminescence was measured using a Victor Nivo plate reader. The IC_50_ was defined as the drug concentration reducing viral RLUs by 50% compared to virus control wells. after subtracting background RLUs. Drug combination effects were calculated as follows:

$$\%\text{ inhibition of HIV}‐1=\frac{\left.\left(1-(\text{Average}\;\text{treatment}\;-\text{Average}\;\text{cell}\;\text{control}\right)\right)}{\left.\left(\text{Average}\;\text{virus control}\;-\text{Average}\;\text{cell}\;\text{control}\right)\right.}\times 100$$

**TABLE 2:** Inhibitory concentrations and cell cytotoxicity of plant extracts against HIV-1 subtype B (NL4.3 and YU2) and HIV-1 subtype C (CM070P.1 and CM019P.1.2) in the TZM-bl cell line.

No.	Plant extracts/ Drugs	Cell cytotoxicity at 50% (CC50) µg/mL	Inhibitory concentration at 50% (IC50) µg/mL				Selective indexes (SI) CC50/IC50			
			NL4.3	CM070P.1	YU2	CM019P.1.2	NL4.3	CM070P.1	YU2	CM019P.1.2
1	Product Nkabinde (PN)	325.5	7.4	0.5	5.97	7.94	44	651	54.5	41
2	Gnidia sericocephala	106.4	219	0.43	959	1.9	0.49	247.4	0.11	56
3	AZT	262.5	19.4	14.1	17.9	23.9	13.5	18.6	14.7	11
4	Raltegravir	124.6	34.6	9.4	31.0	29.8	3.6	13.3	4.0	4.2
5	Maraviroc	226.9	16.5	12.1	4.7	3.3	13.8	18.8	48.3	68.8
6	Amprenavir	104.7	49.0	29.0	26.7	11.0	2.13	3.6	3.9	9.5

*CC50 and IC50 were determined by nonlinear regression from concentration-response curves (Figure 2). SI was calculated by dividing CC50 by IC50.

The fractional inhibitory concentration index (FICI) for the combination treatments was calculated using the following formula:

$$\text{FICI}=\frac{(\text{MICAB})}{\left(\text{MICB}\right)}+\frac{(\text{MICBA})}{\left(\text{MICA}\right)}$$

Where MICAB is the minimum inhibitory concentration (MIC) of drug A tested in combination, MICA is the MIC of drug A tested alone, MICBA is the MIC of drug B tested in combination and MICB is the MIC of drug B tested alone. Values of FICI ≤ 0.5, FICI > 0.5 but ≤ 4.0, and FICI > 4.0 determine synergistic, additive, and antagonistic interactions, respectively.

### 2.12 Statistical analysis

All data were analyzed with GraphPad Prism Version 9.5.1 software (GraphPad Software Inc., San Diego, CA, United States) and presented as mean ± SEM (standard error of the mean). Data are representative of three independent experiments done in triplicates. The corresponding dose-response curves were fitted by non-linear regression analysis using a sigmoidal model. Unless otherwise noted, three independent replicates were assessed for each sample and statistical significance was evaluated using a one-way ANOVA test (Kruskal–Wallis), and p-values ≤0.05 were considered statistically significant.

## 3 Results

### 3.1 *In vitro* cytotoxicity of PN and *G. sericocephala* extracts on PBMCs and TZM-bl cell line

**FIGURE 1:**
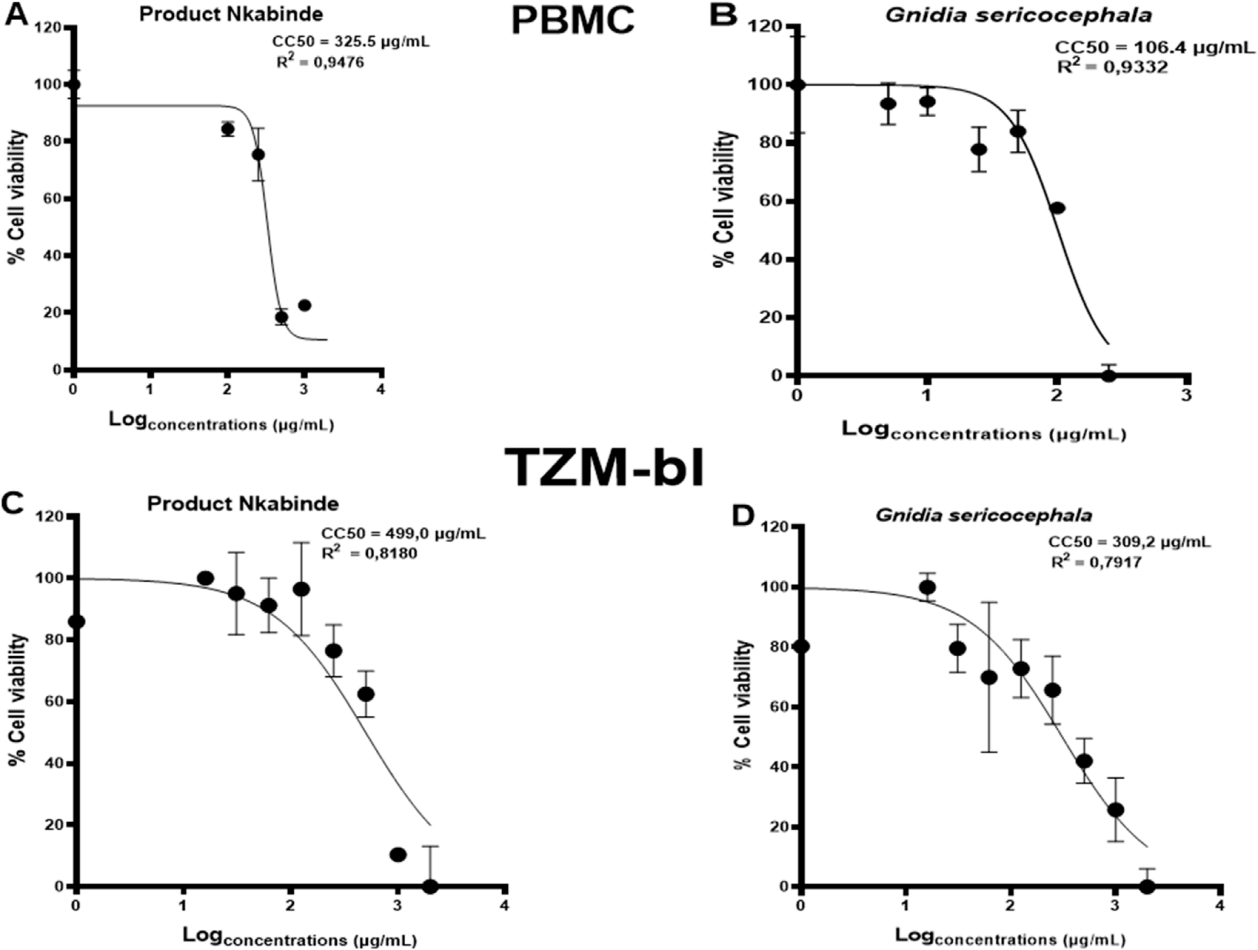
Determination of cytotoxic concentrations of crude extracts in PBMCs and TZM-bl cells. The graphical illustration of dose-dependent curves showing percent viability effects of **(A,B)** PN and *G. sericocephala* on PBMCs and **(C,D)** PN and *G. sericocephala* on TZM-bl cells. The CC50 calculation was done using the GraphPad Prism software.

*In vitro* viability testing of new drug candidates is an essential step in the process of drug discovery and clinical trials (Hughes et al., 2011). The cytotoxicity of each crude extract was evaluated as a percentage of cell viability. PN and *G. sericocephala* were screened for their effects on TZM-bl cells and human PBMC viability using the CellTiter-Glo^®^ (ATP) assay (Table 2). The assay demonstrated dose-dependent cellular viability across a concentration range of 16–2000 μg/mL for PN and 1–250 μg/mL for *G. sericocephala* in both cell types. PN exhibited dose-dependent cytotoxicity with CC50 values of 325.5 μg/mL in PBMCs and 499 μg/mL in TZM-bl cells (Figures 1A,C). Similarly, *G. sericocephala* showed dose-dependent cytotoxicity with CC50 values of 106.4 μg/mL in PBMCs and 309.2 μg/mL in TZM-bl cells (Figures 1B,D). These findings suggest that both extracts exhibit favorable safety profiles as assessed by the CellTiter-Glo^®^ assay.

### 3.2 Antiviral activity of PN and *G. sericocephala* on HIV-1 subtype B and subtype C viruses

**FIGURE 2:**
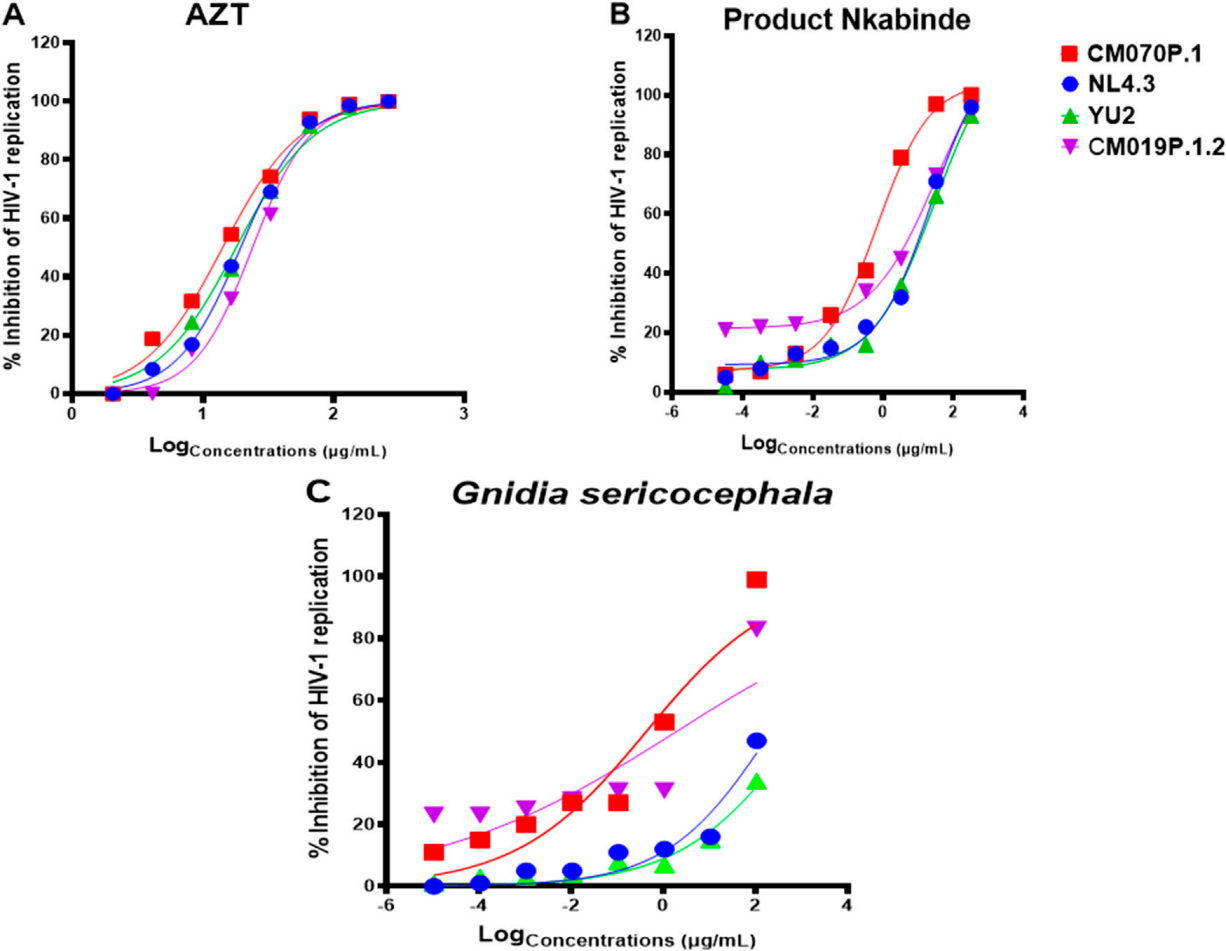
HIV-1 inhibitory effect of PN and G. sericocephala extracts against HIV-1 subtype B and HIV-1 subtype C strains with luciferase-based antiviral assay using TZM-bl cell lines. **(A)** Percentage inhibition of HIV-1 curves of AZT-positive control drug against all four HIV-1 strains. **(B-C)** Percentage inhibition curves of PN and G. sericocephala extract against all four virus strains. The TZM-bl cells were infected with HIV-1 subtype B strains (NL4.3 and YU2) and HIV-1 subtype C strains (CM070P.1 and CM019P.1.2) and treated with the serial dilution of PN and G. sericocephala crude extracts with AZT as a positive control. The infected and treated TZM-bl cells were incubated for 48 hours at 37°C and 5% CO2. The IC50 calculation was done using the GraphPad Prism software. **Viruses: *NL4.3 (blue), CM070P.1 (red), YU2 (green), and CM019P.1.2 (purple)*.

Despite the availability of ART, evaluating medicinal plant extracts for anti-HIV-1 activity remains critical due to limitations such as high cost, toxicity, and the emergence of drug resistance. Plant-based therapies may serve as complementary interventions, especially in resource-limited settings where ART access is restricted, improving treatment efficacy and clinical outcomes (Saehlee et al., 2024; Palshetkar et al., 2020; Kurapati et al., 2016). In this study, the anti-HIV-1 activity of crude plant extracts was assessed using a TZM-bl luciferase assay, which measures LTR-driven luciferase expression as an indicator of viral replication relative to virus-only controls (Tang et al., 2015; Platt et al., 2009). Pseudotype viruses (NL4.3, CM070P.1, YU2, and CM019P.1.2) were only used for single round of infection and allowed us to assess the viral infection levels by measuring the luciferase value in the cell supernatant. As expected, AZT, a licensed antiretroviral drug, effectively inhibited all tested HIV-1 strains (Figure 2A). PN exhibited dose-dependent inhibition across all four viral strains, with maximal inhibition ranging from 93% to 100%. Specifically, PN inhibited NL4.3 by 96% (IC_50_ = 7.4 ± 33.1 μg/mL), YU2 by 93% (IC_50_ = 5.97 ± 40.1 μg/mL), CM070P.1 by 100% (IC_50_ = 0.5 ± 32.1 μg/mL), and CM019P.1.2 by 96% (IC_50_ = 7.94 ± 27.8 μg/mL) (Figure 2B). *G. sericocephala* also demonstrated inhibitory activity against both subtype B and C viruses; however, its effect was generally less potent compared to AZT and PN, with inhibition of CM070P.1 at 99% (IC_50_ = 0.43 ± 30.9 μg/mL), CM019P.1.2 at 83% (IC_50_ = 1.93 ± 21.5 μg/mL), NL4.3 at 47% (IC_50_ = 219.3 ± 15.1 μg/mL), and YU2 at 34% (IC_50_ = 959.4 ± 10.9 μg/mL) (Figure 2C). Taken together these data show that PN extract inhibited the replication of all four viruses, whereas *G. sericocephala* inhibited the replication of HIV-1 subtype C viruses and showed reduced inhibition activity for HIV-1 subtype B viruses. The selectivity index (SI = CC50/IC50) was also calculated to determine the therapeutic index *in vitro*, attaining maximum antiviral activity with minimal cytotoxicity. Thus, PN demonstrated enhanced potency in inhibiting HIV-1 replication for subtype B and C strains, with SI values being 44 (NL4.3), 651 (CM070P.1), 54.5 (YU2), and 41 (CM019P.1.2) (Table 2).

### 3.3 Anti-HIV-1 screening of crude extracts in PBMCs

**FIGURE 3:**
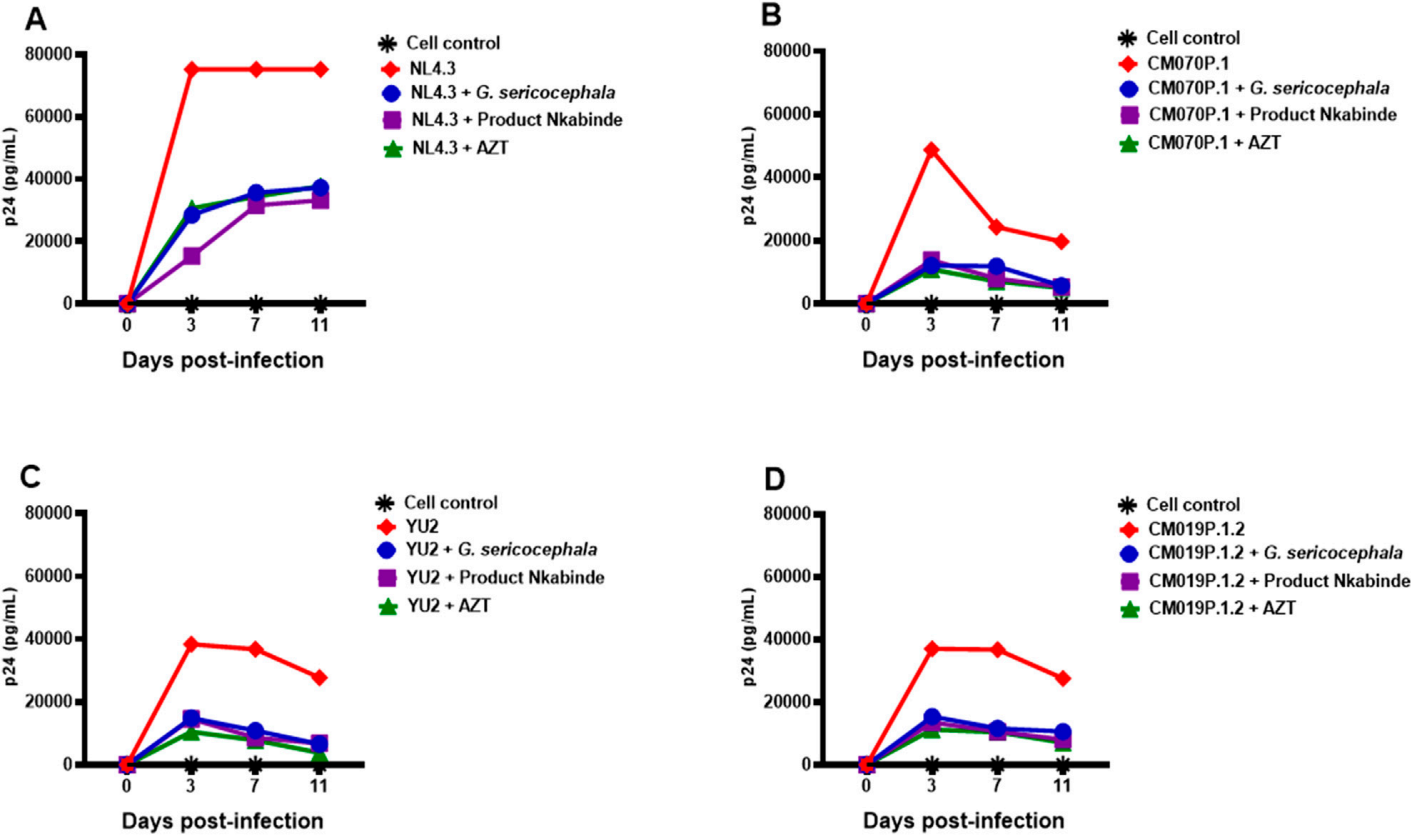
Antiviral activity of crude extracts in PBMCs. **(A–D)** PBMCs activated with PHA and IL-2 for 48 h were incubated in 96-well plates with concentrations of PN (purple), *G. sericocephala* (blue), and AZT (green) and then infected with NL4.3, CM070P.1, YU2 and CM019P.1.2 strains for 72 h. Viral supernatants were collected every 3 days to quantify HIV-1 p24 production using ELISA. Virus controls were denoted by the red color and black asterisks denoted the cell control.

Crude extracts of PN and *G. sericocephala* demonstrated potent inhibition of HIV-1 subtype B and C replication in the TZM-bl luciferase assay. PN, a traditional medicine used by the healer for several years to manage HIV-1 in patients, is typically administered at a dose of three cups per day. Setlhare et al. (2024) reported that PN at 325.3 μg/mL significantly upregulated activation markers and cytokine secretion in healthy PBMCs. To validate the antiviral activity observed in TZM-bl cells, we assessed PN and *G. sericocephala* in PBMCs infected with HIV-1 subtype B (NL4.3, YU2) and subtype C (CM070P.1, CM019P.1.2) strains using the HIV-1 p24 antigen ELISA. AZT was included at its 50% inhibitory concentration for each viral strain (Table 2). Over an 11-day period, PN, *G. sericocephala*, and AZT treatments significantly reduced p24 levels compared to untreated controls, which ranged from 37,000 to 75,000 pg/mL (Figures 3A–D). Partial inhibition was observed for NL4.3, where p24 levels exceeded the 20,000 pg/mL threshold, likely due to suboptimal dosing (Figure 3A). In contrast, p24 levels for CM070P.1, YU2, and CM019P.1.2 remained below 20,000 pg/mL over 7 days, indicating sustained viral suppression (Figures 3B–D). Taken together, these findings highlight the strain-dependent antiviral efficacy of the extracts and AZT in PBMCs.

### 3.4 *In vitro* assessment of crude plant extracts combined with commercial antiretroviral drugs using the TZM-bl cell line

**FIGURE 4:**
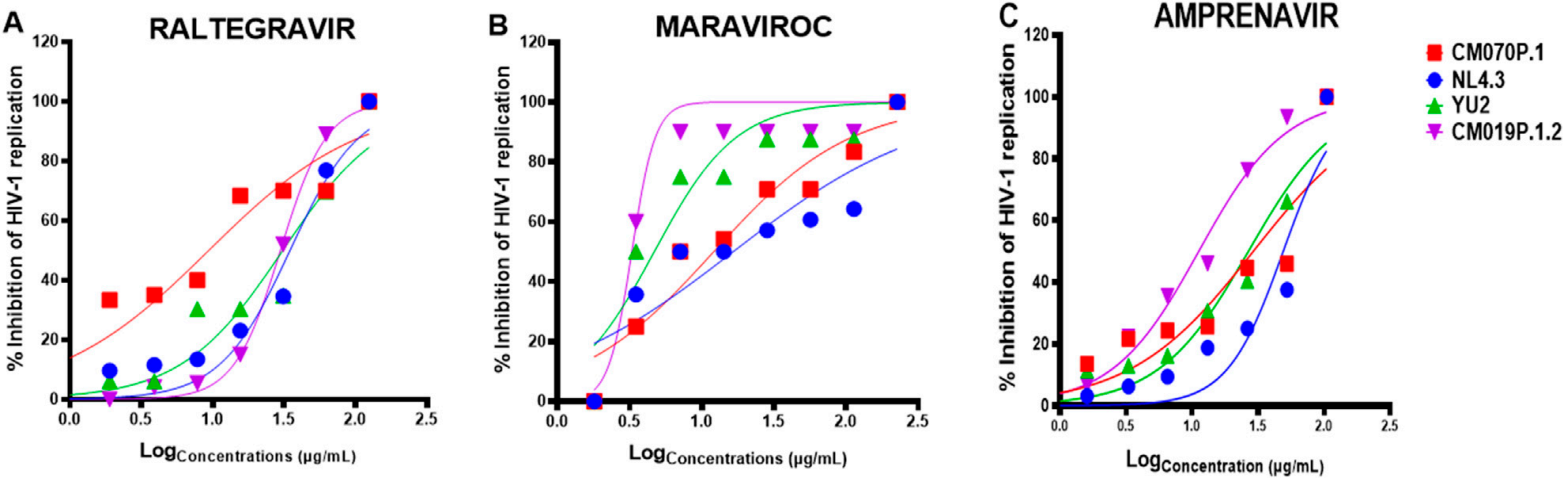
*In vitro* anti-HIV-1 effects of ART drugs. TZM-bl cells were treated with serially diluted concentrations CC50 of **(A)** raltegravir, **(B)** maraviroc, and **(C)** amprenavir and infected with HIV-1B and HIV-1C viral strains for 48 h. The Bright-Glo luciferase assay kit was used for HIV-1 inhibition assay to assess the presence or absence of HIV-1. Key: NL4.3 (blue), CM070P.1 (red), YU2 (green), and CM019P.1.2 (purple).

African Traditional Medicines (ATMs) are commonly used by people living with HIV/AIDS (PLWH), often alongside antiretroviral therapy (ART). However, their clinical efficacy and safety in combination with ARVs remain underexplored. Understanding these interactions is essential for optimizing therapeutic outcomes and minimizing adverse effects, particularly in the context of combination regimens aimed at enhancing antiviral efficacy (Sibanda et al., 2016; Martínez et al., 2014; Park et al., 2014; Lee et al., 2006). To address this gap, we evaluated the cytotoxicity and antiviral efficacy of four antiretroviral drugs including AZT, raltegravir, maraviroc, and amprenavir alone or in combination with traditional medicinal plant extracts (PN and *G*. *sericocephala*) on HIV-1 subtype B (strains NL4.3 and YU2) and HIV-1 subtype C (strains CM070P.1 and CM019P.1.2) in TZM-bl cells. Non-cytotoxic concentrations of each anti-HIV-1 drug were used to assess the anti-HIV-1 effects in TZM-bl cells (Supplementary Figure S1). An antiviral assay was conducted to assess the impact of raltegravir, maraviroc, and amprenavir on HIV-1 replication. As shown in Figures 4A–C, the inhibitory effects of raltegravir, maraviroc, and amprenavir against HIV-1 were ∼37.5–100% and the IC50 values were determined (Table 2), representing 50% inhibition of viral replication of all viruses. Raltegravir, maraviroc, and amprenavir exhibited a dose-dependent inhibition of HIV-1 strains NL4.3, CM070P.1, YU2, and CM190P.1.2 (Figures 4A–C). No cytotoxicity was observed for drug and plant extract combinations in TZM-bl cells (Supplementary Figure S2).

**FIGURE 5:**
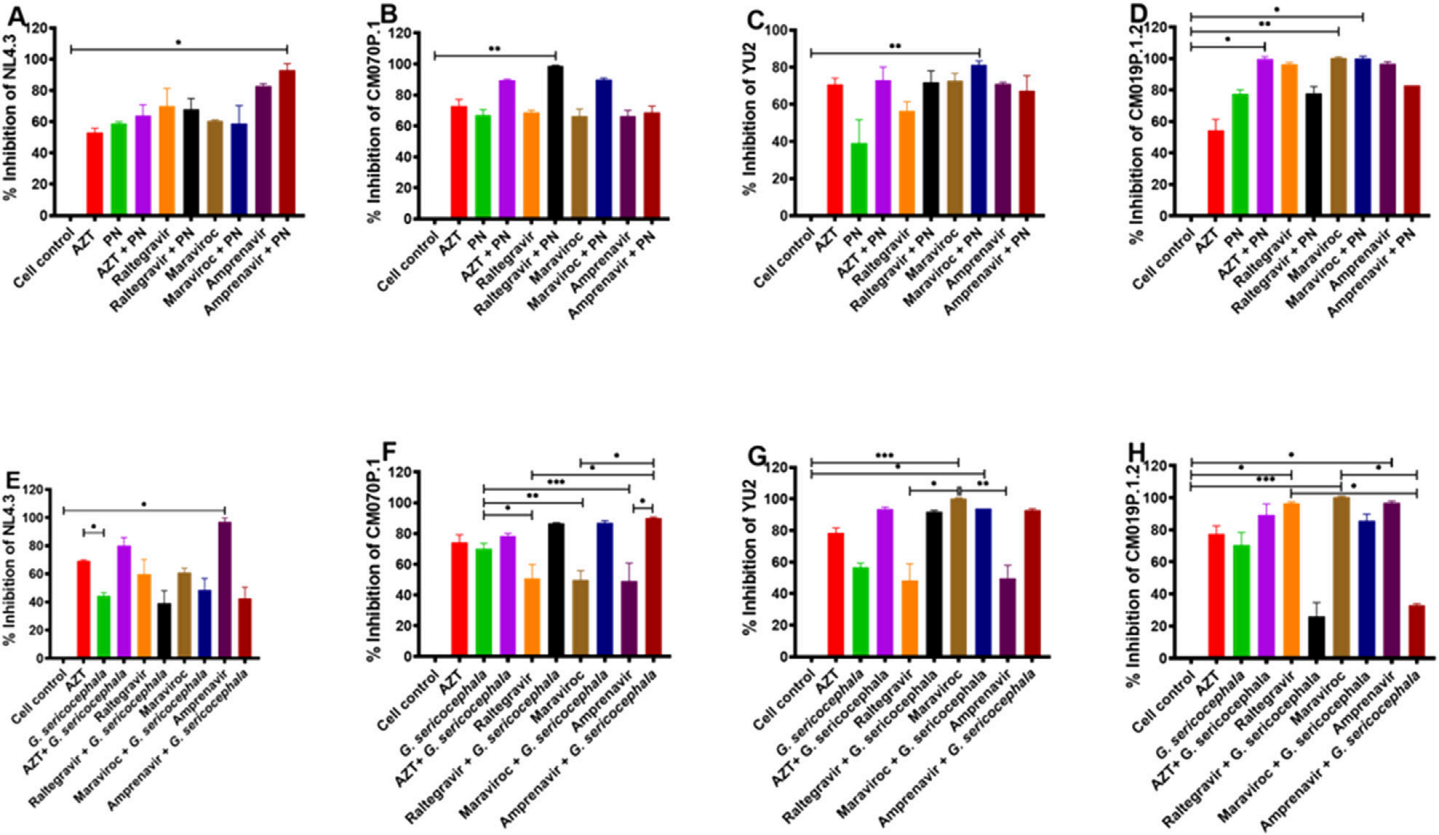
Interactions between crude extracts (PN and *G. sericocephala*) and anti-HIV-1 drugs. **(A–D)** Effect of the anti-HIV-1 drugs (AZT, raltegravir, maraviroc, and amprenavir) and PN treatment combination on TZM-bl cells infected with HIV-1B (NL4.3 and YU2) and HIV-1C (CM070P.1 and CM019P.1.2) viruses for 48 h and the luciferase activity quantify using the plate reader. **(E–H)** Effect of anti-HIV-1 drugs (AZT, raltegravir, maraviroc, and amprenavir) and *G. sericocephala* treatment combination on TZM-bl cells infected with HIV-1B (NL4.3 and YU2) and HIV-1C (CM070P.1 and CM019P.1.2) and the luciferase activity was measured. Different colors represent the various treatment groups. Statistical significance between experimental groups, as calculated by one-way ANOVA, is represented by *p value <0.05; **p value <0.01 and ***p value <0.001.

PN extract combined with AZT exhibited different percentage inhibition of HIV-1 subtype B strain NL4.3 (64%) or amprenavir (93%), while PN extract combined with raltegravir (72%) or maraviroc (81.3%, p = 0.0361) inhibited subtype B strain YU2 (Figures 5A,C). For HIV-1 subtype C, the combination of PN with maraviroc (90%), raltegravir (98.7%, p = 0.0083), and amprenavir (66.6%) inhibited the replication of HIV-1 strain CM070P.1, and PN with AZT (99%, p = 0.0428) inhibited replication of CM019P.1.2 relative to the administration of PN alone (Figures 5B,D). *G. sericocephala* combined with AZT (80.3%, p = 0.0105) significantly inhibited HIV-1 NL4.3 replication. Its combination with AZT (93.7%), raltegravir (92%), and amprenavir (93%) inhibited the replication of HIV-1 YU2 (Figures 5E,G). Additionally, *G. sericocephala* combined with maraviroc (87%, p = 0.0093), raltegravir (86%, p = 0.0168), or amprenavir (90%, p = 0.0006) significantly inhibited subtype C strain CM070P.1, while its combination with AZT (89.3%) inhibited HIV-1 strain CM019P.1.2 relative to *G. sericocephala* and the drugs alone (Figures 5F,H). FICI analysis indicated no synergy, additivity, or antagonism in crude extract and antiretroviral drug combinations. The FICI values for all combinations ranged from 1 to 3.5, indicating an indifferent effect (Supplementary Table S3). In summary, combination of PN and *G. sericocephala* extracts with antiretroviral drugs, showed enhanced inhibition of HIV-1 subtype B and C strains compared to individual treatments.

## 4 Discussion

The effectiveness of combination antiretroviral therapy (cART) is constrained by factors such as adverse drug reactions, the emergence of resistant viral strains, long-term drug toxicity, high treatment costs, the viral reservoir, and suboptimal patient adherence (Terefe et al., 2022; Clercq, 2001). Furthermore, some people living with HIV-1 still have limited access to treatment in low-income countries. To address these shortcomings, it is necessary to identify locally available, less expensive, and less toxic HIV-1 treatment options to treat this disease. Nature has always been an important source for treating different ailments and diseases. Previously published reviews have reported that several medicinal plants have been found to have anti-HIV-1 properties (Terefe et al., 2022; Cos et al., 2004; Yang et al., 2001). In addition, various secondary metabolites derived from natural sources demonstrated moderate to good anti-HIV-1 activity, with some showing excellent activity (Cos et al., 2004; Jung et al., 2000; Matthée et al., 1999).

This study investigated the potential anti-HIV-1 activity of African traditional medicine, specifically, Product Nkabinde (PN) and *G. sericocephala*, by evaluating their ability to inhibit HIV-1 replication in the TZM-bl cell line and human PBMCs. The TZM-bl cell line, derived from HeLa cells, was engineered via retroviral transduction to express CD4, CXCR4, and CCR5, making it highly permissive to HIV-1, SIV, and SHIV infection (Morozov et al., 2022; Platt et al., 1998). Additionally, these cells are transfected with lentiviral vectors encoding firefly luciferase and *E. coli* β-galactosidase reporter genes under HIV-1 LTR control (Kappes and Wu, 2005; Montefiori, 2004; Wei et al., 2002). Upon infection, viral reverse transcription and cDNA integration lead to Tat-mediated activation of reporter gene expression. The luciferase activity, quantified by luminescence, directly correlates with infectious virus levels, while β-galactosidase activity can be measured through X-gal staining assays. This system enables a robust assessment of viral replication and the efficacy of candidate natural products (Morozov et al., 2022). PBMCs were also utilized because they incorporate a degree of physiologic relevance, a useful system for studying viral behaviour, host-virus interactions, and therapeutic interventions. These cells naturally express HIV-1 entry co-receptors including CCR5, CXCR4 (Wieczorek et al., 2013).

*In vitro* viability testing of new drug candidates is an essential step in the process of drug discovery and clinical trials. To ensure the safety of PN and *G. sericocephala* extracts, cytotoxicity tests were conducted using PBMCs and TZM-bl cells and quantified using the Celltiter-Glo™ ATP assay. The CellTiter-Glo^®^ assay measures ATP, a key indicator of metabolically active cells, therefore the luminescence produced is proportional to the number of viable cells in a given sample (Lugongolo et al., 2021). Based on our results, increased concentrations of PN and *G. sericocephala* demonstrated low levels of ATP production, indicating a decreased number of viable cells compared to the untreated control. A CC50 value higher than the control suggests that higher concentrations of crude extracts are required for the cytotoxic effect to appear. According to the United States National Cancer Institute Plant Screening Program, a crude extract is considered to have *in vitro* cytotoxicity when the CC_50_ value is <30–40 μg/mL (Talib and Mahasneh, 2010). Therefore, the cytotoxicity results indicate that the crude extracts showed no cytotoxicity on TZM-bl cells and human PBMCs. Tembeni et al. (2022) reported that four extracts (n-hexane, dichloromethane, ethyl acetate, and methanol) obtained by the sequential extraction of *G. sericocephala* roots were screened using HeLa-SXR5 cells demonstrated no overt cytotoxicity using the *in vitro* decipher assay.

It has been reported that botanicals like *Rheum palmatum* L., *Rheum officinale, Trigonostem axyphophylloides, Vatica astrotricha, Vernonia amygdalina Hypoxias pelargonium, Sidoides hemerocallidea,* and *Sutherlandia frutescens,* have been reported to possess anti-HIV-1 activity (Rakshit et al., 2024; Huerta-Reyes et al., 2021; Tshikalange et al., 2008). The phytoconstituents isolated from these plants are important in HIV-1 inhibitory activity. Our study used a TZM-bl luciferase-based assay to assess the anti-HIV-1 activity of crude extracts PN and *G. sericocephala*. Our findings from the antiviral assay revealed that PN showed potent anti-HIV-1 activity against both HIV-1 subtype B (NL4.3 and YU2) and subtype C (CM070P.1 and CM019P.1.2) viral strains, comparable to AZT. *G. sericocephala* demonstrated an increased percentage of HIV-1 subtype C viral inhibition, while subtype B strains showed low percentages of HIV-1 inhibition. The potent antiviral activity of PN and *G. sericocephala* extract may be attributed to bioactive compounds present in the medicinal plants used in its formulation. UPLC-HRMS analysis by Tembeni et al. (2022) and Setlhare et al. (2024) confirmed the presence of bioactive daphnane-type diterpenes, including yuanhuacine A, gniditrin, yuanhuajine, and yuanhuacine, in both PN and *G*. *sericocephala*. These compounds have been reported to exert antiviral effects by downregulating CXCR4, a coreceptor critical for HIV-1 entry into host cells, thereby disrupting early stages of the viral life cycle (Tembeni et al., 2022; Hou et al., 2021; Vidal et al., 2012). Moreover, Vonranke et al. (2022) demonstrated through molecular docking studies that certain diterpenes from marine sources can bind to allosteric sites on HIV-1 reverse transcriptase, inducing conformational changes that may inhibit viral RNA binding. While these findings pertain to marine-derived compounds, they support the plausibility of similar mechanisms for plant-derived daphnane diterpenes and warrant further mechanistic investigation (Vonranke et al., 2022).

We further validated these results in PBMCs using the HIV-1 p24 ELISA assay and the findings suggested that PN and *G. sericocephala* have potent anti-HIV-1 activity comparable to AZT even when human primary cells were used. Tembeni et al. (2022) observed this with the isolated compound yuanhuacine A, which was tested in PBMCs infected with a subtype C viral strain. The yuanhuacine A compound demonstrated antiviral activity comparable to AZT, with an EC50 and EC90 of 0.03 and 0.09 µM, respectively (Tembeni et al., 2022). In addition, the selectivity index (SI) is a fundamental parameter for distinguishing true antiviral activity from cytotoxic effects. A high SI value confirms that viral inhibition is not a consequence of cellular toxicity (Barros et al., 2012; Tamargo et al., 2015; Muller and Milton, 2012). Our results indicate that the PN extract exhibited SI values exceeding 10, demonstrating greater selectivity for viral inhibition compared to the AZT control. Furthermore, PN showed the highest SI values among all tested viruses compared to *G. sericocephala*. Antiviral activity is considered significant when IC_50_ values are below 100 μg/mL (Cos et al., 2006). Therefore, the IC_50_ and SI values of the PN extract meet these criteria, supporting its potential for investigation in preclinical models. Similarly, Terefe et al. (2022) reported that *Croton megalocarpus* leaf extract and *Croton dichogamus* aerial part extract demonstrated high antiviral activity with low cytotoxicity and high SI values compared to AZT in MT-4 cells. Additionally, an aqueous extract from *Guettarda angelic* seeds exhibited potent anti-herpesvirus activity with high SI values (Barros et al., 2012).

People living with HIV/AIDS (PLWH) frequently use African traditional medicines (ATM), either alone or in combination with Western medicines, including antiretroviral therapy (ARTs) to treat HIV-1-related symptoms and side effects (Sibanda et al., 2016; Martínez et al., 2014; Lee et al., 2006). However, herbal medicines may cause clinically significant interactions with antiretroviral agents, potentially leading to treatment failure (Blonk et al., 2012). ATMs are often complex mixtures of organic compounds, which can activate and/or inhibit the enzymatic pathways that are involved in the metabolism of antiretroviral drugs. The activation and/or inhibition of these metabolic enzymes that clear antiretroviral drugs may decrease their concentrations, leading to reduced efficacy and drug failure. On the contrary, inhibiting these metabolic enzymes can result in the elevation of ART concentrations and intensify their toxicity (Lee et al., 2006). Despite the widespread use of combination therapy, further research is necessary to determine the safety and efficacy of combining ATMs with ARTs to improve clinical outcomes and understand pharmacokinetics. In clinical practice, it is important to investigate the combinations of several drugs that boost the antiviral activity of available drugs while suppressing their unwanted side effects (Park et al., 2014). To explore the potential impact of combination treatments between ARTs, PN, and *G. sericocephala*, the antiretroviral drugs that were used in this study belong to four classes of ARTs, namely, protease inhibitor (PI) (amprenavir), integrase inhibitor (raltegravir), CCR5 entry inhibitor (maraviroc) and nucleoside reverse transcriptase inhibitor (NRTI) (AZT). The efficacy of these ART drugs to reduce HIV-1 infection individually was demonstrated by dose-dependent inhibition curves after the addition of ART drugs to the infected cells. This was followed by an evaluation of the anti-HIV-1 effects of ARTs in combination with PN and *G. sericocephala*. Results showed that the combination of crude extracts and ART drugs did not aggravate cytotoxicity to TZM-bl cells at concentrations used in the current experiments. We then used the fractional inhibitory concentration index (FICI) to assess how combination treatments interacted to exert anti-HIV-1 activity. Our results indicate that crude extract combinations with antiretroviral drugs exhibited positive efficacy interactions for PN, whereas *G. sericocephala* alone demonstrated antagonistic interactions with select anti-HIV-1 drugs, including raltegravir, maraviroc, and amprenavir. However, no synergy, addictive effect, or antagonism was found for the combinations tested. Drugs with the same target often show additive or antagonistic interactions (Calzetta and Koziol-White, 2021; Niu et al., 2019; Tekin et al., 2016).

Limitations in the current findings include the fact that while the current results demonstrate promising anti-HIV-1 activity of PN and *G. sericocephala*, *in vitro* findings do not always translate to *in vivo* efficacy due to differences in drug metabolism, bioavailability, and immune system interactions. In addition, while antiviral activity was observed, the specific molecular mechanisms underlying the effects remain unclear. Further studies are necessary to elucidate potential interactions with viral replication pathways. Future research should focus on *in vivo* studies, clinical trials, and mechanistic investigations to determine the therapeutic potential and safety of this traditional medicine product in HIV-1 treatment.

## 5 Conclusion

Exploring ATMs for their anti-HIV-1 properties reveals a rich potential for developing new antiviral therapies. The combination of low cytotoxicity and effective inhibition of HIV-1 replication in laboratory settings by PN and *G. sericocephala* suggests that further research could lead to viable treatment options derived from these natural sources. Continued investigation into the specific compounds responsible for these effects will be crucial in harnessing their full therapeutic potential. In addition, since the combination results showed no negative interactions, more research is needed that will focus on the clinical outcome of dual therapy. Furthermore, while the FICI method provides an initial understanding of potential combinatory effects, more detailed analyses including dose-response matrices and isobologram-based models can be used to better characterize these drug interactions.

## Data Availability

The original contributions presented in the study are included in the article/Supplementary Material, further inquiries can be directed to the corresponding author.
