# Supplementary material for: Herbal formulations, Product Nkabinde and Gnidia sericocephala, exhibit potent in vitro activity against HIV-1 infection
Source: Front Pharmacol. 2025 Jul 2;16:1618187. doi: 10.3389/fphar.2025.1618187 (PMC12264436; doi:10.3389/fphar.2025.1618187)
Supplement: Supplementary file 1 [file Supplementaryfile1.pptx]

## Slide 1
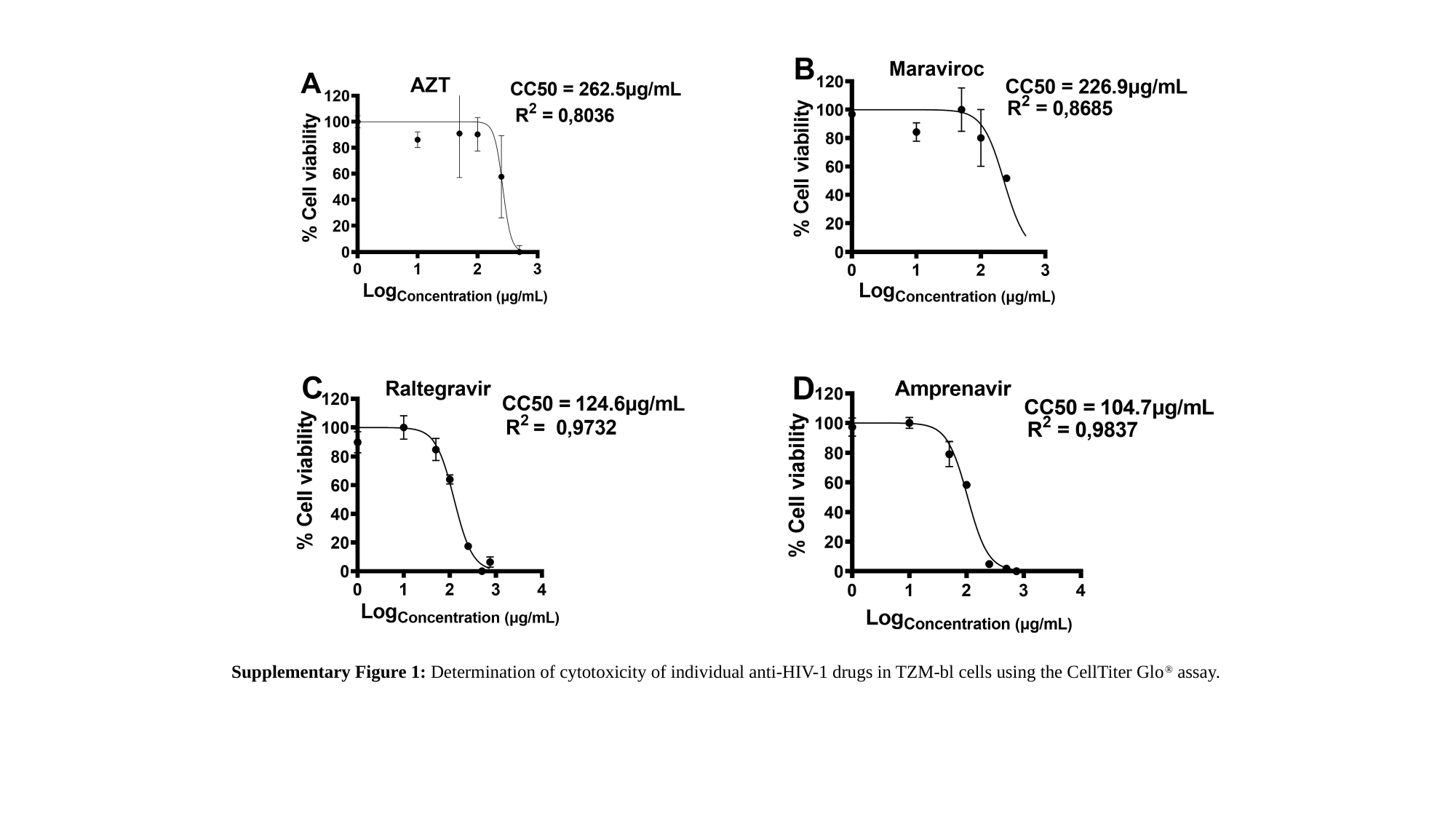

Supplementary Figure 1: Determination of cytotoxicity of individual anti-HIV-1 drugs in TZM-bl cells using the CellTiter Glo® assay.

## Slide 2
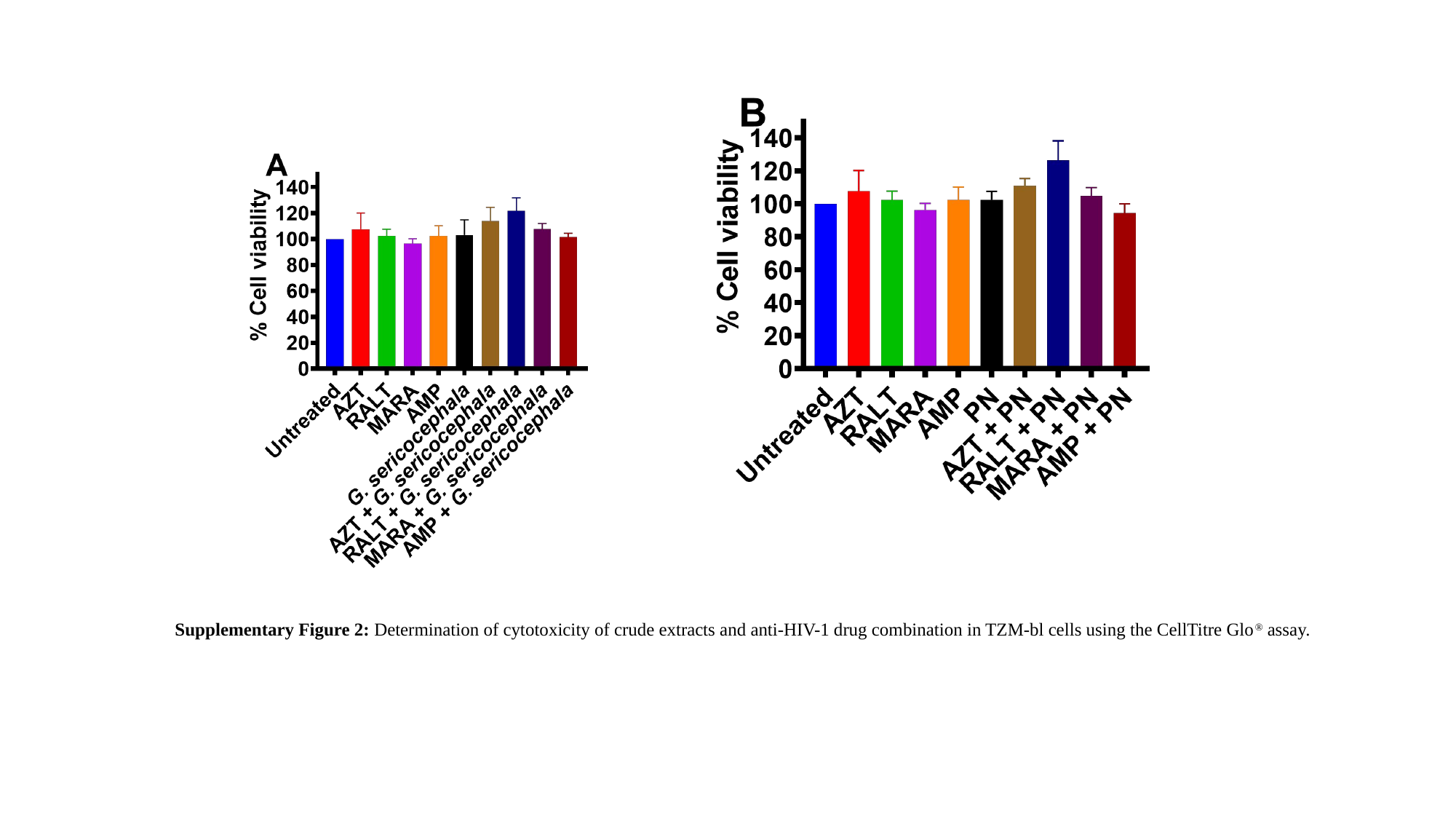

Supplementary Figure 2: Determination of cytotoxicity of crude extracts and anti-HIV-1 drug combination in TZM-bl cells using the CellTitre Glo® assay.

## Slide 3
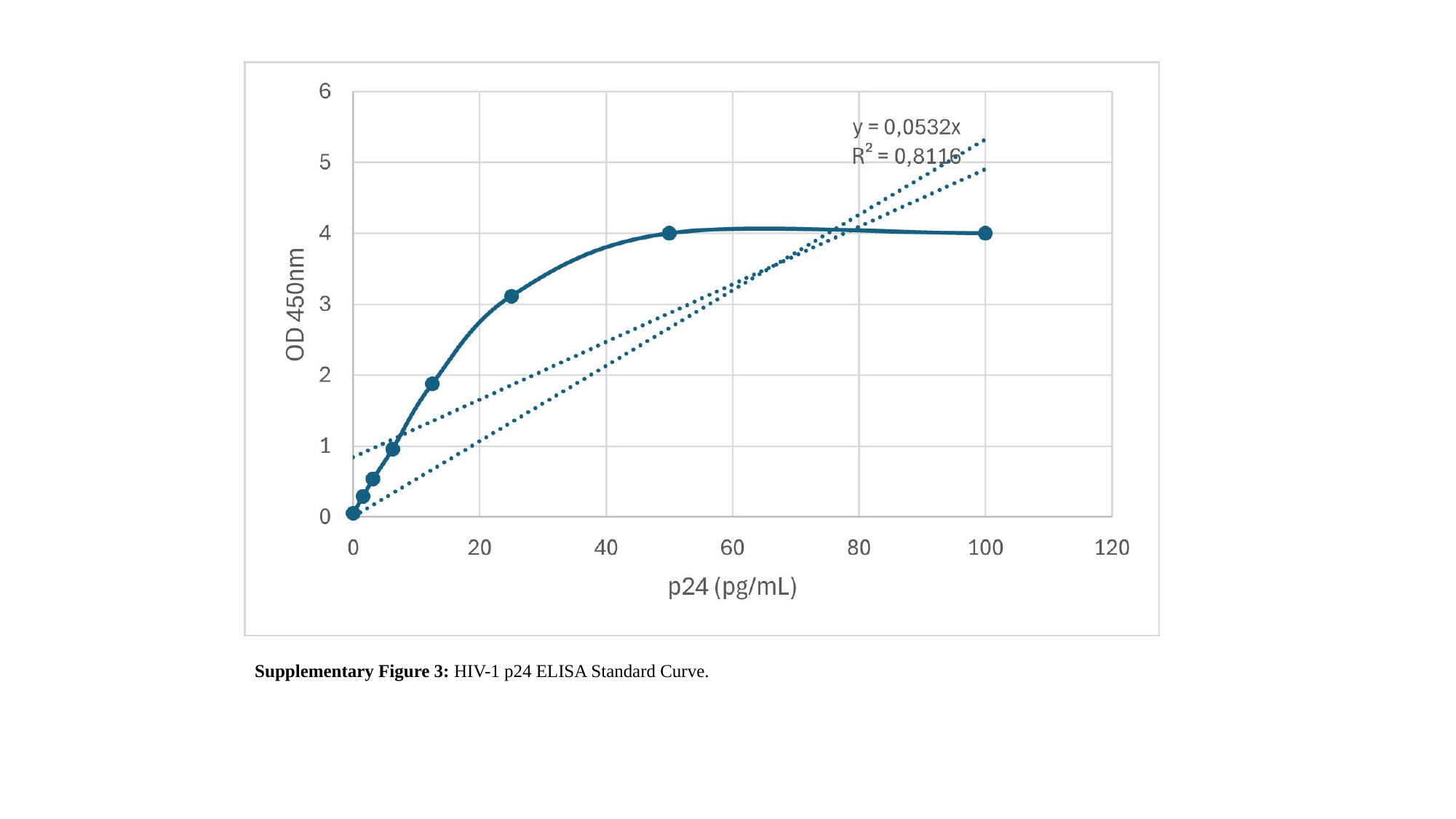

Supplementary Figure 3: HIV-1 p24 ELISA Standard Curve.

## Slide 4
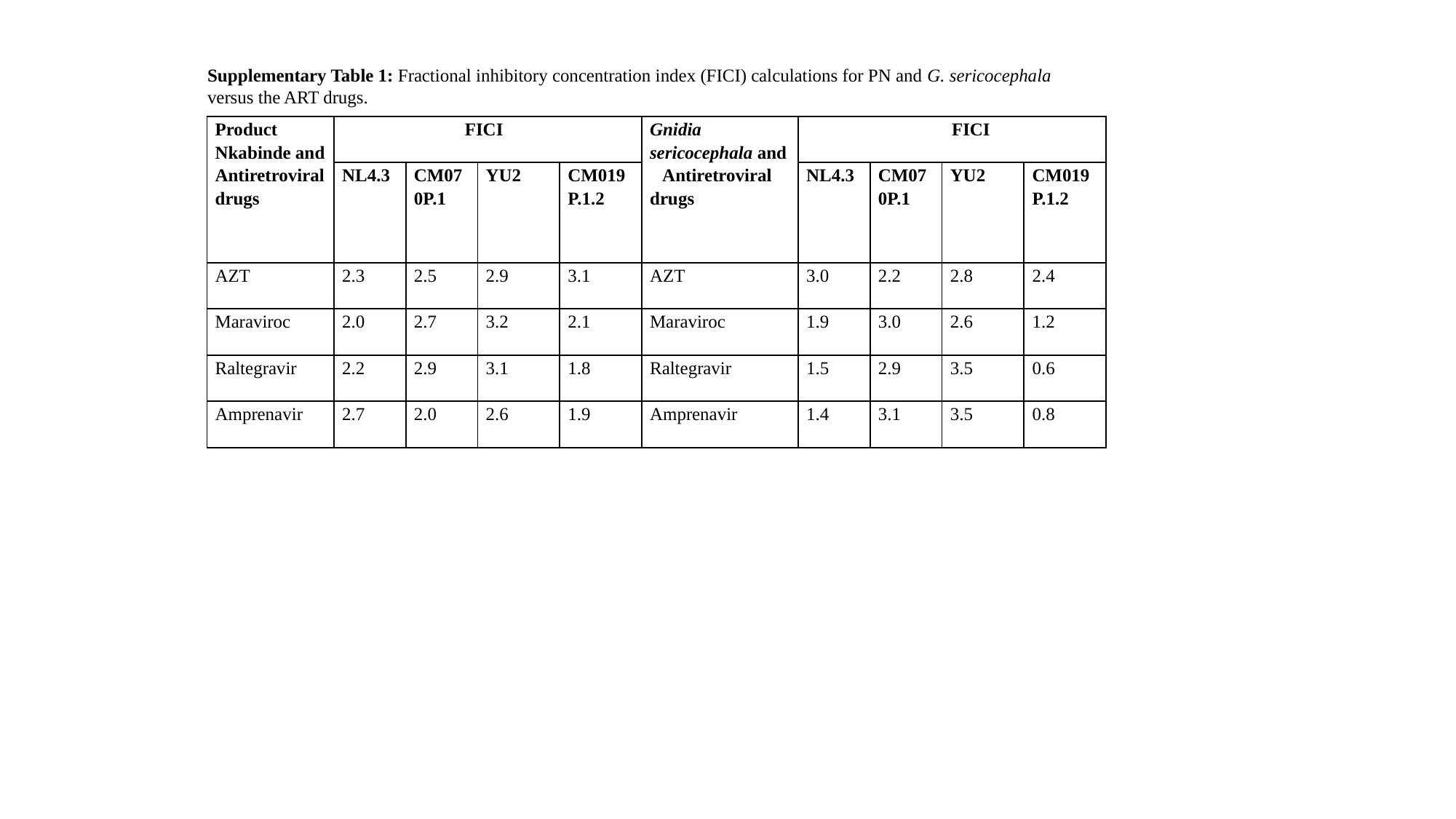

Supplementary Table 1: Fractional inhibitory concentration index (FICI) calculations for PN and G. sericocephala
versus the ART drugs.
| Product Nkabinde and Antiretroviral drugs | FICI | | | | Gnidia sericocephala and Antiretroviral drugs | FICI | | | |
| --- | --- | --- | --- | --- | --- | --- | --- | --- | --- |
| | NL4.3 | CM070P.1 | YU2 | CM019P.1.2 | | NL4.3 | CM070P.1 | YU2 | CM019P.1.2 |
| AZT | 2.3 | 2.5 | 2.9 | 3.1 | AZT | 3.0 | 2.2 | 2.8 | 2.4 |
| Maraviroc | 2.0 | 2.7 | 3.2 | 2.1 | Maraviroc | 1.9 | 3.0 | 2.6 | 1.2 |
| Raltegravir | 2.2 | 2.9 | 3.1 | 1.8 | Raltegravir | 1.5 | 2.9 | 3.5 | 0.6 |
| Amprenavir | 2.7 | 2.0 | 2.6 | 1.9 | Amprenavir | 1.4 | 3.1 | 3.5 | 0.8 |
